# Comparative Gas Sorption and Cryoporometry Study of Mesoporous Glass Structure: Application of the Serially Connected Pore Model

**DOI:** 10.3389/fchem.2019.00230

**Published:** 2019-04-16

**Authors:** Henry R. N. B. Enninful, Daniel Schneider, Antonia Hoppe, Sandra König, Michael Fröba, Dirk Enke, Rustem Valiullin

**Affiliations:** ^1^Faculty of Physics and Earth Sciences, Felix Bloch Institute for Solid State Physics, Leipzig University, Leipzig, Germany; ^2^Faculty of Chemistry and Mineralogy, Institute of Chemical Technology, Leipzig University, Leipzig, Germany; ^3^Institute of Inorganic and Applied Chemistry, University of Hamburg, Hamburg, Germany

**Keywords:** mesoporous glasses, structural characterization, pore size analysis, freezing and melting, gas sorption, disorder, serially-connected pore model

## Abstract

Nitrogen sorption and melting and freezing of water in a small pore size mesoporous glass with irregular pore structure is studied. The analysis of the experimentally obtained data is performed using the recently developed serially connected pore model (SCPM). The model intrinsically incorporates structural disorder by introducing coupling between nucleation and phase growth mechanisms in geometrically disordered mesopore spaces. It is shown that, in contrast to the independent pore models prevailing in the literature, SCPM self-consistently describes not only boundary transitions, but also the entire family of the scanning transitions. The scanning behavior is shown to be very sensitive to microscopic details of the fluid phase distribution within the porous materials, hence can be used to check the validity of the thermodynamic models and to improve the structural analysis. We show excellent quantitative agreement between the structural information evaluated from the cryoporometry and gas sorption data using SCPM.

## 1. Introduction

Mesoporous solids offer good balance between their pore size, relatively high specific surface area, and beneficial transport properties. This makes them attractive for many potential applications in chemistry, medicine, and energy storage (Mal et al., 2003; Čejka and Mintova, 2007; Popat et al., 2011; Fried et al., 2013; Moller and Bein, 2013; Perego and Millini, 2013; Pal and Bhaumik, 2015). It is often that optimization of their various properties, such as catalytic activity, transport rate, and separation efficiency are needed for specific applications. One of the strategies in this respect is purposeful tuning the geometry of the pore space. Designing the pore structure requires, in turn, a set of tools allowing to establish the resulting pore architecture (Mitchell et al., 2012; Coasne et al., 2013; Cychosz et al., 2017). Among others, gas sorption is most widely used for structural analysis of mesoporous solids (Thommes et al., 2015). This method relies on the confinement effects upon gas-liquid and liquid-gas transition points for fluids in the mesopore spaces. Another alternative approach, generally referred to as cryo- or thermoporometry, is also widely used (Brun et al., 1977). In complete analogy with the gas sorption, this method relies on the measurements of the solid-liquid and liquid-solid transition points. The two methods have a lot in common and may be treated in a similar way.

On considering mesoporous solids with ideal pore structures, substantial progress in understanding the fluid phase equilibria in these materials has been made over the last decades (Evans and Tarazona, 1984; Evans, 1990; Ravikovitch et al., 2001; Neimark et al., 2003; Monson, 2012). With the development of NLDFT methods the phase state in single pores can nowadays be predicted with very high accuracy. The challenging point is to rationalize how accurate these predictions will be if the pore geometry starts deviating from having ideal shape, for example that of a perfect cylinder. So far, the approaches existing and applied to porous solids with complex pore morphologies assume that these materials can be represented as a superposition of single pores in which phase transitions do not interfere with each other. In this way, the complex structure is subdivided into independent single pores and the global phase equilibrium is found as the sum over individual elements using, for example, the general adsorption isotherm (Thommes et al., 2015). This phenomenology is often referred to as the independent pore or independent domain model (IPM).

One of the main assumptions made in the frame of IPM is that phase transformations, such as condensation or evaporation, in pores with different sizes constituting the entire pore network occur independently from each other. As a consequence, these models operate with the relationships unambiguously relating the thermodynamic variable, temperature *T* or pressure *p*, and the critical pore size. This information is normally encoded in the so-called kernels, which are the family of curves showing phase composition in a pore with a certain pore size as a function of either temperature or pressure. Irrespective of the fact whether a transition is considered to occur at thermodynamic equilibrium or governed by the barriers in the free energy, there will be a monotonic variation of the critical pore sizes with *T* or *p*. Hence, for a given transition pathway and at given *T* and *p*, there will be one single, well-defined pore size, dividing the pore size distribution into two parts. Namely, below and above this critical pore size fluid in the pores will be in different phase states. This is a direct consequence of the fact that in IPM phase equilibria in different pores are not interrelated.

At the same time, it is commonly accepted that there are physical mechanisms giving rise to interference between the phase states in adjacent pores with different pore sizes (Cordero et al., 2002; Ravikovitch and Neimark, 2002; Morishige and Tateishi, 2003; Libby and Monson, 2004; Rigby and Fletcher, 2004; Morishige et al., 2006; Casanova et al., 2007; Khokhlov et al., 2007; Rasmussen et al., 2010; Reichenbach et al., 2011; Nguyen et al., 2013b; Kondrashova and Valiullin, 2015; Mitropoulos et al., 2015; Rigby et al., 2017). One of them is pore blocking, namely metastability arising from the fact that the invasion of the gas or ice phase is delayed until it can pass a bottleneck on its way. Hence, the phase growth process in disordered materials becomes a function of how pores with different sizes are connected to each other. In the same spirit, advanced sorption or advanced melting, resulting from coupling of nucleation and phase growth processes, render spatial distribution and interconnectivity important for the progress of phase transformations. Note that they are irrelevant for IPM.

The most simple scenario on how to address the cooperativity effects in phase transitions is to model the pore spaces of real porous solids by one dimensional chains of single pores with different pore sizes, in what follows referred to as serially connected pore model (SCPM). The idea comes from early studies of an ordered MCM-41 silica material with cylindrical pores in mesopore range (Kruk et al., 1997; Liu et al., 2000). Nitrogen sorption isotherms measured in this material with the shapes untypical for ordered materials were interpreted to be related to disorder in the pore structure (Coasne et al., 2006). Using 3-D electron tomography, such defects were directly visualized in a material with similar pore morphology, SBA-15 (Gommes, 2012). By using numerical analysis of computer generated 3-D disordered network models in combination with advanced sorption and pore blocking, it was shown that, accounting for geometric disorder, leads to qualitatively correct shapes of the sorption isotherms and, most importantly, of the scanning curves (Liu et al., 1993; Cordero et al., 2002; Esparza et al., 2004). Later, by considering truly SCPM, namely computer generated chains of disordered tubular pores, and by modeling either only pore blocking (Coasne et al., 2005) or both pore blocking and advanced sorption (Morishige, 2017), it was shown that already this simplistic model is capable of reproducing all important features of the three-dimensional models. Note that modeling in all these studies was essentially deterministic, i.e., the phase transition behavior was assessed based on a set of rules for the cooperativity mechanisms and afterwards averaged over different disorder realizations. Microscopic details of how these effects emerge in long chains were explored using combinations of only few pore sections using GCMS simulations (Nguyen et al., 2013a). Analogous studies, but with notably longer chains, have proven the emergence of cooperativity effects and validated the conclusions made earlier (Kondrashova and Valiullin, 2013, 2015; Schneider et al., 2017).

Being effective in providing better understanding of the phase equilibria in geometrically-disordered mesopore spaces, computer-generated models are not suitable for the structural analysis. Hence, some attempts to formulate the respective thermodynamic theoretical models have been made. In the studies by Mason (1982, 1983, 1988) and Cimino et al. (2013), a notable progress was made by capturing the real 3-D pore structure using the Bethe model. Another approach is based on the lattice gas models on random graphs (Handford et al., 2014) or disordered matrices (Doan et al., 2014). On the other hand, as has been mentioned in the preceding paragraph, the phase behavior in SCPM exhibits all properties of 3-D models. Moreover, because of the simplicity of SCPM, it turns out that the problem of phase transitions with included cooperativity effects can be solved analytically, namely, the transition equation (e.g., isotherm in the context of capillary condensation phenomena) can be derived. The first formulation of the theoretical model related to SCPM (Kondrashova et al., 2016) and its solution (Schneider et al., 2017) have been presented recently. In this work, we show the first application of the SCPM theory for structural analysis of a disordered mesoporous silica material. First, we recapitulate the most important points of SCPM. Thereafter, we apply SCPM for the analysis of freezing and melting of water and of nitrogen sorption in the material studied. Two different experimental approaches we purposefully use to provide additional evidence to the robustness of the SCPM theory.

## 2. Serially Connected Pore Model

For the sake of simplicity, the subsequent description is intentionally done for the capillary condensation and evaporation transitions. One should note, however, that the same phenomenology is applied for other phase transitions too. The only modification which is needed is simple replacement of the governing microscopic kernels describing the phase transitions involved. Let us start with the general adsorption isotherm, which is the core of the IPM-based approaches,

(1) $$\begin{array}{l} \theta {\left(p\right)}_{IPM}=\int \theta_{i}\left(p,x\right)\phi \left(x\right)dx \end{array}$$

In Equation (1), ϕ(*x*) is the normalized pore size distribution (PSD), θ(*p, x*)_*i*_ is a kernel, *i* referring to a particular kernel (typically metastable or equilibrium). To recall, for any thermodynamic condition there will be single well-defined critical pore diameter *x*_*cr*_ dividing ϕ(*x*) into two parts. Thus, above *x*_*cr*_ the pores contain liquid film and gas in the core and below *x*_*cr*_ all pores are completely filled with the condensed liquid. In SCPM, the filling or emptying of the pores is a combination of two effects, namely, nucleation and phase growth. Hence, Equation (1), containing only one of these modes, does not apply anymore and needs to be revised.

In SCPM, the pore network is modeled as a linear chain of *L* joined cylindrical pore sections with identical section lengths, *l*, and pore diameters, *x*, distributed according to a PSD ϕ(*x*). It is assumed that the pore diameters for two adjacent pore sections are not correlated, i.e., statistical disorder is implied. Upon increase and decrease of the gas pressure, the phase transition in a selected pore section can occur by nucleation of a new phase or by phase growth if the new phase is already provided in the section adjacent to the considered one. On desorption we consider gas invasion (phase growth) and cavitation (nucleation), while on adsorption liquid bridging (nucleation) and advanced sorption (phase growth). In the spirit of Equation (1), we seek for the solution in the form

(2) $$\begin{array}{l} \theta_{\text{ads}}\left(p,x\right)=\int \theta_{g}\left(x,p\right)\psi \left(x,p\right)dx+\int \theta_{n}\left(x,p\right)\psi^{\prime }\left(x,p\right)dx, \end{array}$$

and

(3) $$\begin{array}{l} \theta_{\text{des}}\left(p,x\right)=\int \theta_{n}^{\prime }\left(x,p\right)\psi \left(x,p\right)dx+\int \theta_{g}\left(x,p\right)\psi^{\prime }\left(x,p\right)dx. \end{array}$$

Here ψ(*x, p*) and ψ′(*x, p*) denote the PSDs of the filled and empty sections, respectively. Note the prime character refer to the section not containing capillary condensed liquid. Just by the definitions of ψ and ψ′, it is evident that ψ(*x, p*) + ψ′(*x, p*) = ϕ(*x*). The indices “n” and “g” in these equations refer to the kernels as determined by only nucleation and by only growth transition mechanisms, respectively. It turns out that with the kernels it is convenient to model the amounts adsorbed in the empty and filled pore sections in analog to Equation (1). In this way, the adsorption transition can be written as the integral of the sum of two products of (i) the equilibrium kernel θ_*g*_(*x, p*) and the PSD of the filled sections and of (ii) the metastable liquid bridging kernel θ_*n*_(*x, p*) and the PSD of the empty sections. Similarly, for the desorption transition one has to consider two products of (i) the equilibrium kernel θ_*g*_(*x, p*) with the PSD of the empty sections and of (ii) the metastable kernel $\theta_{n}^{\prime }\left(x,p\right)$ with the PSD of the filled sections. An approximate solution for Equations (2) and (3) is presented in the work by Schneider et al. (2017) and the exact derivation can be found elsewhere (Schneider and Valiullin, submitted). In what follows, we present the final results.

In Equations (2) and (3), the only unknown quantities are the distributions ψ and ψ′. By referring to the fact that in SCPM the chains are statistically disordered, the distributions ψ and ψ′ can be obtained exactly using the combinatorial analysis. It can be shown that the distribution ψ(*x, p*) is a piece-wise function

(4) $$\begin{array}{l} \psi \left(x,p\right)=\phi \left(x\right)\times \{\begin{array}{cc} 1 & x\leq x_{n}\\ P_{tr} & x_{n}<x\leq x_{g},\\ 0 & x_{g}<x \end{array} \end{array}$$

where *x*_*n*_ and *x*_*g*_ are the critical pore diameters for liquid bridging and advanced sorption, respectively, and *P*_*tr*_ is a coefficient determining the fraction of the pore sections in which the capillary condensed liquid was formed due to advanced sorption. This quantity is obtained to be

(5) $$\begin{array}{l} P_{tr}={\left(P_{g}-P_{n}\right)}^{-1}\left(P_{g}\frac{\sum_{1}^{L}P_{\text{st}}\left(\lambda \right)\lambda f_{g}\left(\lambda \right)}{\sum_{1}^{L}\lambda f_{g}\left(\lambda \right)}-P_{n}\right), \end{array}$$

where

(6) $$\begin{array}{l} P_{\text{st}}\left(\lambda \right)=1-{\left(1-\frac{P_{n}}{P_{g}}\right)}^{\lambda } \end{array}$$

and

(7) $$\begin{array}{l} f_{\text{g}}\left(\lambda \right)=\frac{1}{P_{g}^{2}+LP_{g}-LP_{g}^{2}}\{\begin{array}{cc} P_{g}^{\lambda }\left(1-P_{g}\right)\left[2+\left(L-\lambda -1\right)\left(1-P_{g}\right)\right] & \lambda <L\\ P_{g}^{L} & \lambda =L \end{array} \end{array}$$

The probability *P*_*n*_, for a nucleation event to occur in a randomly selected pore section, and the probability *P*_*g*_, that a randomly selected pore section can be filled with the capillary condensed liquid if at least one of its adjacent pore sections already contains condensed liquid, are given by

(8) $$\begin{array}{l} P_{n}\left(p\right)=\frac{\int_{x}^{x_{n}}x^{-2}\phi \left(x\right)dx}{\int_{0}^{\infty }x^{-2}\phi \left(x\right)dx} \end{array}$$

and

(9) $$\begin{array}{l} P_{g}\left(p\right)=\frac{\int_{x}^{x_{g}}x^{-2}\phi \left(x\right)dx}{\int_{0}^{\infty }x^{-2}\phi \left(x\right)dx}, \end{array}$$

respectively. These probabilities have earlier been introduced by Mason (1988) and are the functions of the thermodynamic conditions, in this case of pressure *p*. The desorption branch is treated in the same way, only the cavitation and gas invasion probabilities need to be redefined accordingly. As well, the boundary condition accounting for gas invasion from channel openings need to be incorporated.

The same strategy is applied to obtain the desorption scanning curves as covered in the present study. Exactly the same set of the equations used to calculate the boundary desorption transition are applied. The only difference is that, now, one has to introduce the initial conditions differing from that used for the boundary desorption transition. Thus, for addressing the boundary desorption transition we have considered a chain of length *L*, in which initially all sections were filled with the capillary condensed liquid. For the scanning desorption transition, however, the initial condition is given by a distribution of the domains with capillary condensed liquid alternating with the domains containing only liquid film. The distribution of the domain lengths λ_*d*_ for the domains filled with the condensed liquid, *f*(λ_*d*_), can be easily found for any point along the adsorption boundary transition as

(10) $$\begin{array}{l} f\left(\lambda_{d}\right)=\frac{P_{\text{st}}\left(\lambda \right)f_{g}\left(\lambda \right)}{\sum_{\lambda =1}^{L}P_{\text{st}}\left(\lambda \right)f_{g}\left(\lambda \right)}. \end{array}$$

With this distribution function, the final solution for the desorption scan is obtained by ensemble average over Equation (3) in which *L* is replaced by λ_*d*_.

## 3. Transition Kernels

As it has been seen in the preceding section, SCPM is responsible for predicting the global behavior. In Equations (2) and (3), the SCPM framework yields the distributions ψ and ψ′. The microscopic properties of the system, on the other hand, are captured by the transition kernels. For the analysis of the experimental data presented further on, it is essential therefore to describe the kernels which have been used for the subsequent analysis. While the kernels for gas sorption were extensively addressed in the literature, this concept is rarely covered for freezing and melting. Hence, we start first with a detailed discussion of how the kernels for the latter transitions were compiled in our work and then we shortly mention which literature sources were used for obtaining the sorption kernels.

### 3.1. Freezing and Melting Transitions

To obtain the transition kernels one needs two components. First, when a pore contains frozen ice, between the ice core and the pore walls t a non-frozen liquid layer (NFL) forms (Overloop and Vangerven, 1993). Its thickness τ is found to be a function of temperature. Thus, NFLs will contribute to the amount of the liquid phase found in the pore space. In our work, we modeled the thickness τ(*T*) following the work by Liljeblad et al. (2017), where accurate values for τ obtained for D_2_O on a silica surface are provided. We further assumed that for H_2_O the same dependency will be observed if one considers identical temperature suppression *T*_0_ − *T* from the bulk transition point *T*_0_.

To model the freezing and melting transitions we used the modified Gibbs-Thompson equation

(11) $$\begin{array}{l} T_{0}-T=\frac{a\gamma \upsilon T_{0}}{\Delta H}\frac{1}{x-2\tau \left(T\right)}, \end{array}$$

where γ = 30 mJ/m^2^ is the surface free energy of the solid-liquid interface, υ = 18.1 cm^3^/mol the molar volume, Δ*H* = 6 kJ/mol the latent heat of melting, and *x* pore diameter. The parameter *a* is a pre-factor defined by the pore geometry and the transition mechanism as described in what follows.

**Figure 1:**
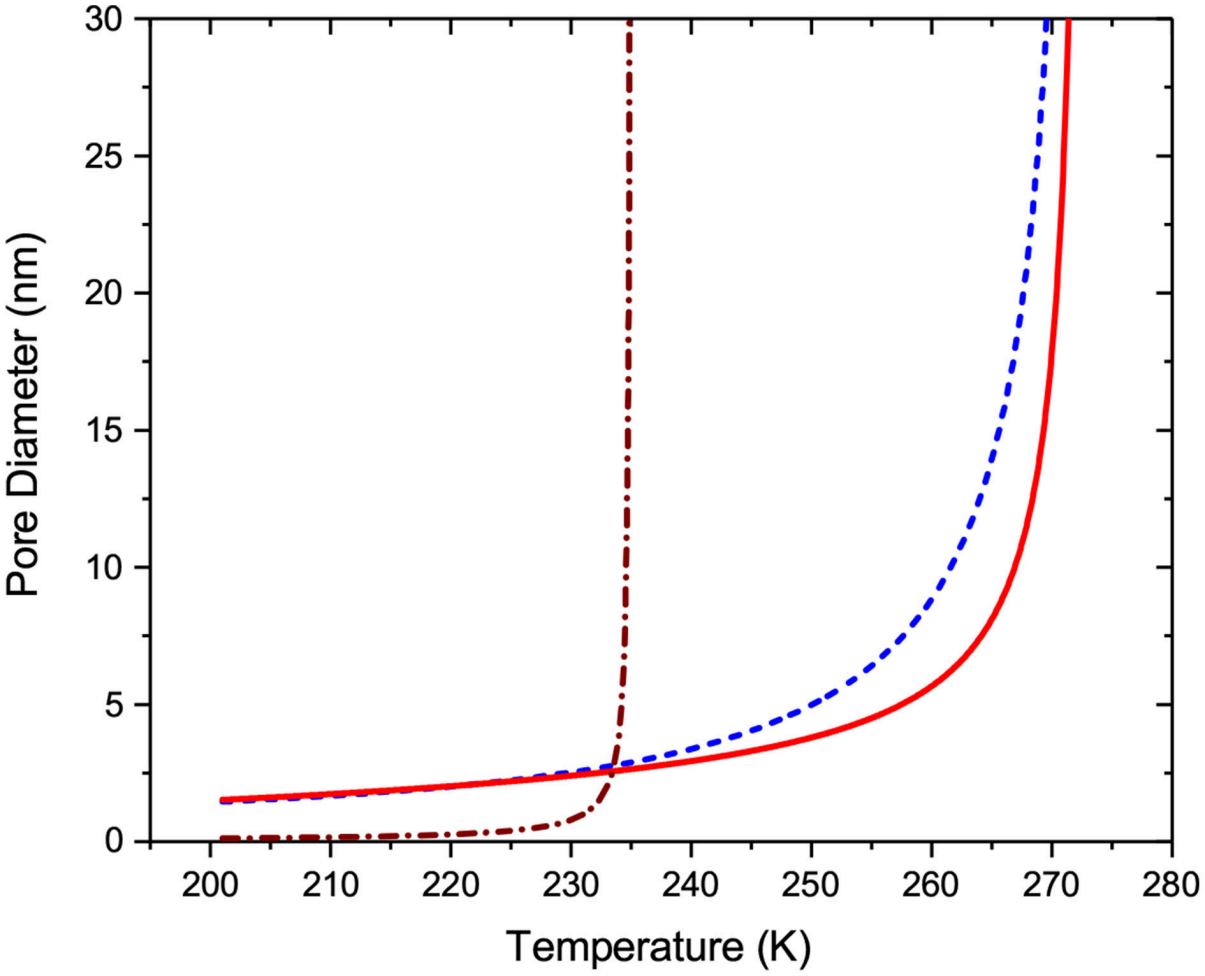
Critical pore diameters for different freezing and melting transition mechanisms: The broken line (blue) refers to the equilibrium transition, the solid line (red) to the metastable liquid bridging, and the dotted-broken line (brown) to the homogeneous ice nucleation.

The cryoporometry experiments are typically performed with excess liquid provided. To study the freezing behavior, first the sample is cooled down, so that the excess water freezes. Afterwards, temperature is increased to a temperature slightly below *T*_0_. In this way, it is assured that, prior to the freezing experiments, the frozen ice phase is provided at the pore openings. With this boundary conditions fixed, the main freezing mechanism is the ice invasion from the pore openings. The phase transition thus takes place at equilibrium and, for cylindrical pores, is described by Equation (11) with *a* = 4. The respective curve is shown in Figure 1. It may happen that relatively large pores in the material interior are connected to the pore openings via smaller pores in which either no crystallization can take place or the equilibrium transition temperature in them is lower than *T*_*h*_ ≈ 233*K* (Mascotto et al., 2017). The latter is known as a homogeneous nucleation temperature below which liquid state of water cannot be sustained anymore and supercooled water spontaneously turns into ice (Amann-Winkel et al., 2016). Analogous to the cavitation pressure in confined fluids (Rasmussen et al., 2010), *T*_*h*_ may be pore size dependent. Establishing this relationship is, however, a challenging problem for future studies. In this work, we have introduced a very weak dependency using a phenomenological equation *T*_*h*_ − *T* = 2/*d*. This will not introduce any notable effect on the data analysis, but will render the freezing transition controlled by homogeneous ice nucleation not abrupt and visually smoother (see Figure 1).

Upon warming, two melting mechanisms are considered: (i) by nucleation of liquid bridges and (ii) by growth of the already formed liquid domains. Melting in a cylindrical pore of a finite length with the liquid phase provided at the pore openings will occur at thermodynamic equilibrium. Hence, it may be modeled by Equation (11) with *a* = 4 and coincides with the one for equilibrium freezing. The second mechanism presumes metastability of the frozen phase, hence is modeled with Equation (11) with *a* = 2 as for infinitely long cylindrical pores. This, however, is true only for relatively wide pores. Experimental and computer modeling studies provided the evidence that hysteresis between freezing and melting diminishes with decreasing pore size (Jahnert et al., 2008). This is caused by increasing impact of thermodynamic fluctuations with decreasing pore size (Kondrashova and Valiullin, 2015) and which are completely ignored in the derivation of the Gibbs-Thompson equation. Eventually, this results in the elimination of metastability for sufficiently small pores. Because to date there is no clear understanding of how to handle these fluctuation effects, in this work the liquid bridging was modeled as a smooth transition from the behavior governed by Equation (11) with *a* = 2 (metastable melting) for large pores to that governed by Equation (11) with *a* = 4 (equilibrium melting) for small pores. In the latter case it is assumed that thermodynamic fluctuations in small pores efficiently eliminate metastability rendering it close to equilibrium transition. The respective curve is shown in Figure 1.

**Figure 2:**
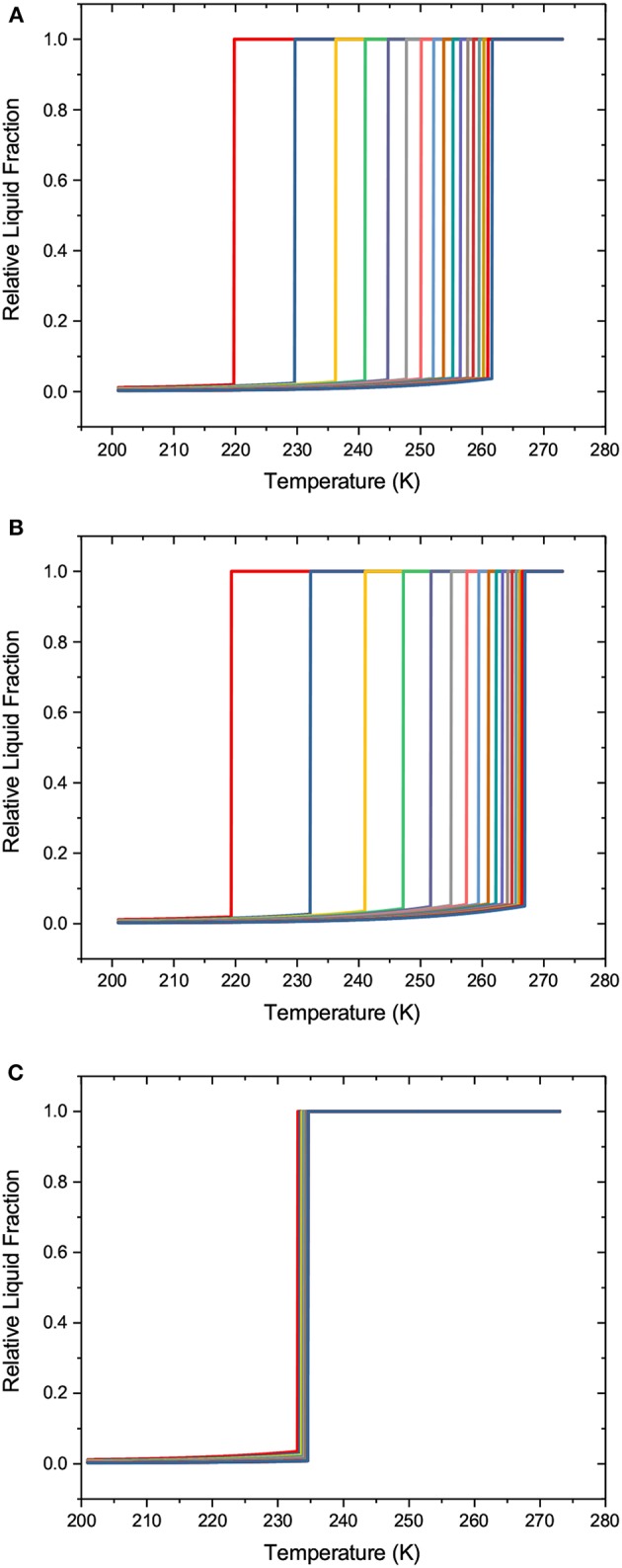
The freezing and melting transition kernels: **(A)** for equilibrium melting and freezing, **(B)** for metastable melting, and **(C)** for homogeneous ice nucleation. Different lines refer to different pore diameters from 2 to 10 nm (from left to right) in steps of 0.5 nm.

Now, by combining the transition temperatures for different transition mechanisms with the thickness of NFLs, one may compile a set of the transition kernels which will be used for the subsequent analysis of the transition behaviors using SCPM. These kernels are shown in Figure 2. Note that because the equilibrium transition points are identical for equilibrium freezing (ice invasion) and equilibrium melting (growth of liquid domains), finally only three kernels are used.

### 3.2. Adsorption and Desorption Transitions

**Figure 3:**
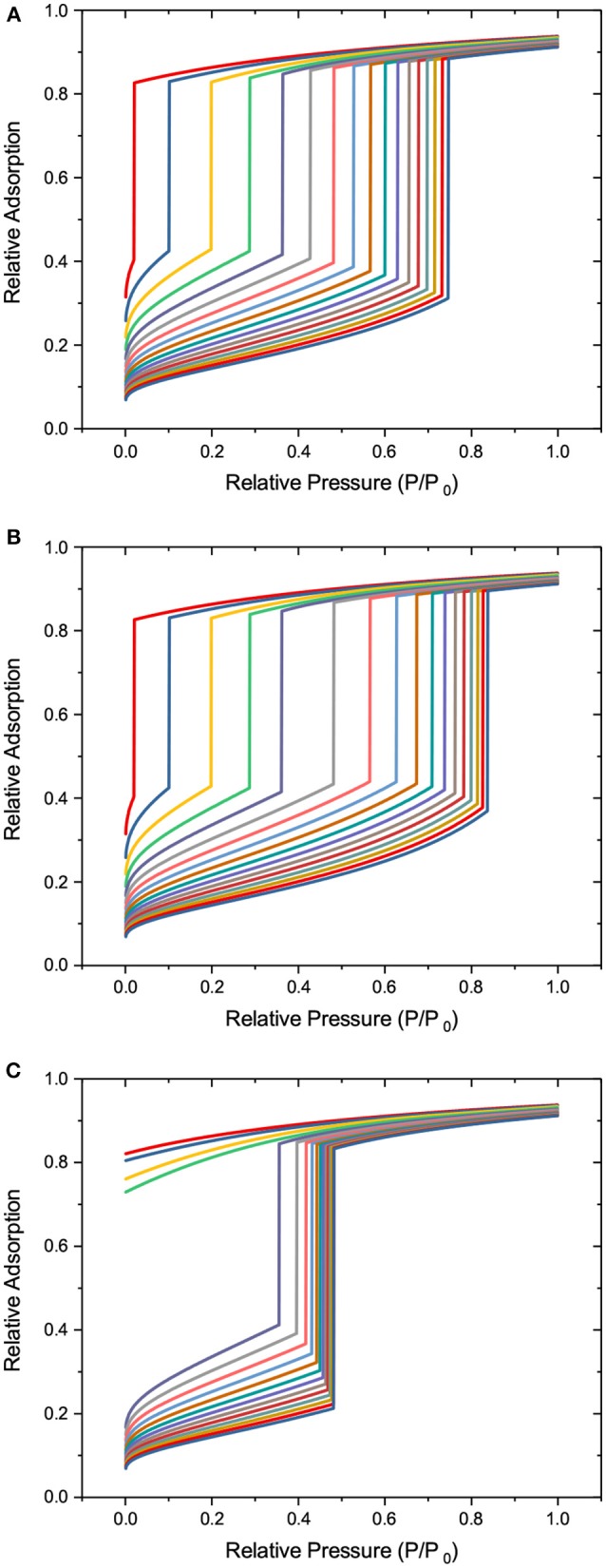
The sorption transition kernels: **(A)** for equilibrium adsorption and desorption, **(B)** for metastable adsorption, and **(C)** for cavitation. Different lines refer to different pore diameters from 2 to 10 nm (from left to right) in steps of 0.5 nm.

**Figure 4:**
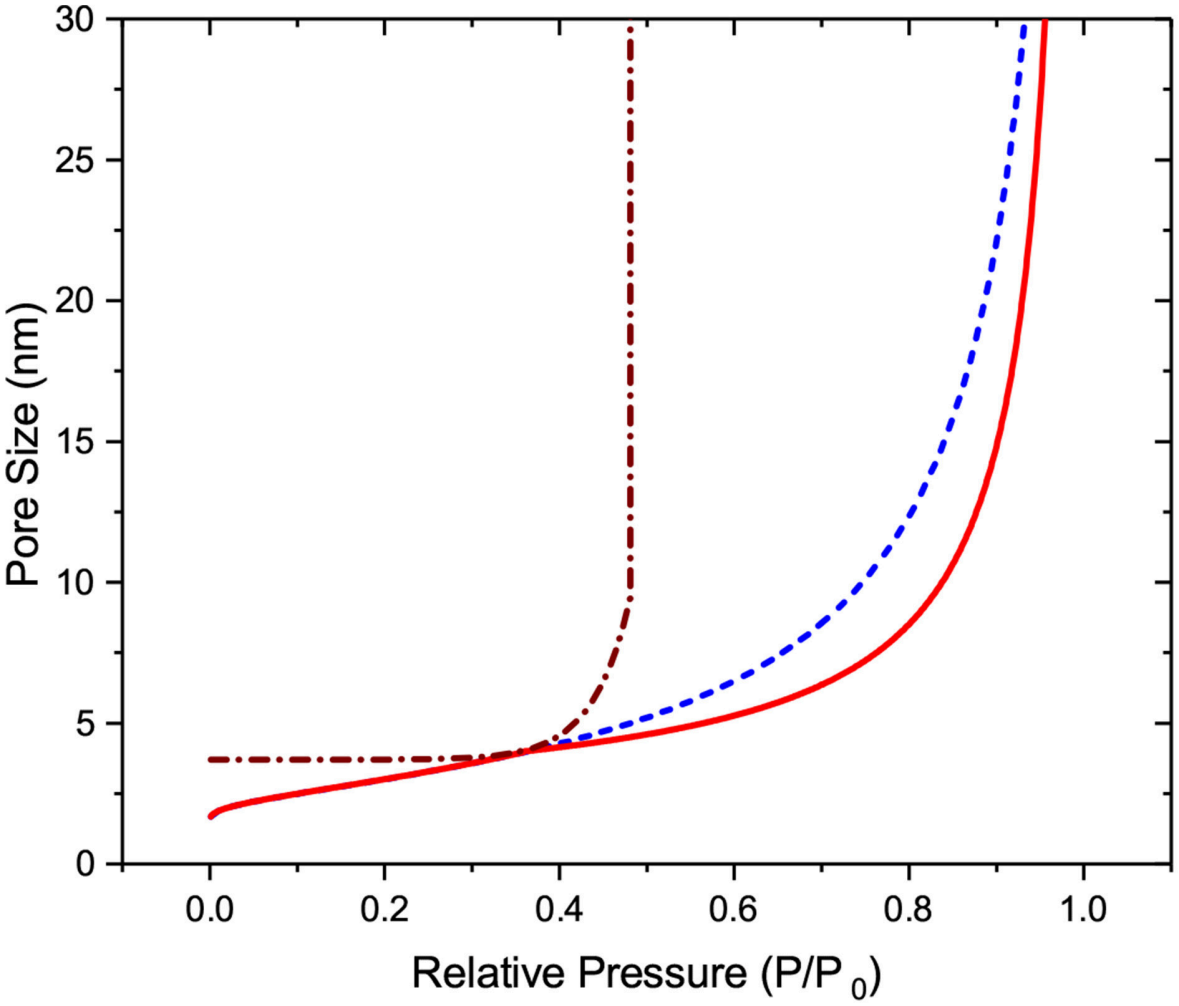
Critical pore diameters for different sorption transition mechanisms: The broken line (blue) refers to the equilibrium transition, the solid line (red) to the metastable liquid condensation, and the dotted-broken line (brown) to cavitation.

In contrast to melting and freezing, sorption of gases by porous solids is extensively addressed in the literature. The kernels used in our work are shown in Figure 3. They are combinations of the Harkins-Jura equation for the adsorbed film thickness δ

(12) $$\begin{array}{l} \delta \left[nm\right]=0.1{\left(\frac{13.99}{0.034-\log\left(p/p_{0}\right)}\right)}^{0.5} \end{array}$$

with the functions relating the critical pore diameters and the equilibrium (Neimark et al., 2003), metastable (Neimark et al., 2003), and cavitation (Rasmussen et al., 2010) transition pressures as shown in Figure 4.

## 4. Materials and Methods

### 4.1. Porous Glass

Mesoporous glass beads with grain sizes between 80 and 100 μm were used as model system. The materials were prepared according to the VYCOR process via phase separation and selective leaching. In the first step, non-porous glass beads with a composition of 70 wt.% SiO_2_, 23 wt.% B_2_O_3_, and 7 wt.% Na_2_O were heat treated for 12 h at 813 K (heating ramp 10 K/min) to initiate the phase separation. In the next step, the surface sealing (SiO_2_-rich dense layer) was removed from the outer surface of the beads by treatment with 6 N NaOH at 333 K for 6 min. The soluble phase was then leached with 3 N HCl at 363 K for 15 h. The obtained mesoporous glass beads were washed with deionized water until a neutral pH value was reached and finally dried at 393 K prior to use.

### 4.2. NMR Cryoporometry

NMR cryoporometry (Brun et al., 1977; Overloop and Vangerven, 1993; Strange et al., 1993; Petrov and Furó, 2009) was employed over a temperature range between 220 and 270 K. The NMR experiments were performed with a cryo-magnet operating at 2.35 T. As a probe liquid, de-ionized water with bulk transition temperature *T*_0_ of 273.15 K was used. Water was preferred over other liquids since its thermodynamic properties are extensively studied and are obtained independently. The proton (^1^H) NMR signal was measured using a Hahn echo sequence with an inter-pulse delay τ = 0.3 ms between the two radio frequency pulses. This delay chosen for our experiments was sufficiently longer than the transverse relaxation time *T*_2_ of the order of 10 μs (Valiullin and Furó, 2002) in frozen water phase, hence, ensured that the signal measured were from the liquid phase only and ice phase did not contribute. This implies that the spin-echo amplitudes measured correlated with the liquid phase fraction in the sample at each temperature step.

As prepared, the porous glass sample was first placed in a clean, dry test tube, outgassed for 24 h using a turbomolecular pump and oversaturated with water under vacuum. It was ensured the presence of excess bulk water around the materials. Temperature was slowly reduced to completely freeze out water in pores and then increased to about 270 K to ensure that only the bulk water phase was completely frozen, while intra-pore fluid remained in the liquid sate. The frozen bulk phase at the pore opening on the material outer boundaries acted as nuclei to initiate freezing, thereby eliminating uncontrolled super-cooling effects. Afterwards, temperature was changed stepwise with each reduction of temperature accompanied by an intermittent temperature equilibration of 5 min until barely any signal change could be seen. Similarly, melting was proceeded with temperature in the reverse direction. For detailed understanding of the network effects, freezing scanning measurements were performed by slowly freezing out intra-pore liquid after partially melting some part of the frozen water at 243 and 249 K.

To ensure reliable data for our studies, the NMR signal amplitudes were corrected for both nuclear magnetic relaxation and Curie (the proportionality between magnetization and the inverse of temperature) effects. By directly measuring the transverse (*T*_2_) relaxation times of intra-pore supercooled water during the cooling process, correction of the signal intensities obtained was performed. More details on the experimental procedure may found elsewhere (Mitchell et al., 2008). By repeating the NMR cryoporometry experiments several times, we have proven that the results were reproducible.

### 4.3. Nitrogen Sorption

Physisorption isotherms were measured with a Micromeritics 3Flex Surface Analyzer. The porous glass sample was activated prior to the measurement using a Micromeritics SmartVac Prep Degasser with turbomolecular pump. Degassing procedure was conducted under vacuum. The sample cell was heated up with a ramp of 5 K/min to 393 K followed by an isothermal segment of 20 h. Hysteresis scanning measurements were performed with nitrogen as adsorptive at 77 K. Liquid nitrogen was used as coolant. The hysteresis loop was scanned with six equally distributed desorption isotherms. After measuring an initial isotherm, the pore structure was partially filled again followed by a high resolution desorption measurement. For each scan, the degree of pore filling was decreased in 0.025 *p*/*p*_0_ steps.

## 5. Experimental Results and Discussion

### 5.1. Freezing and Melting of Water

**Figure 5:**
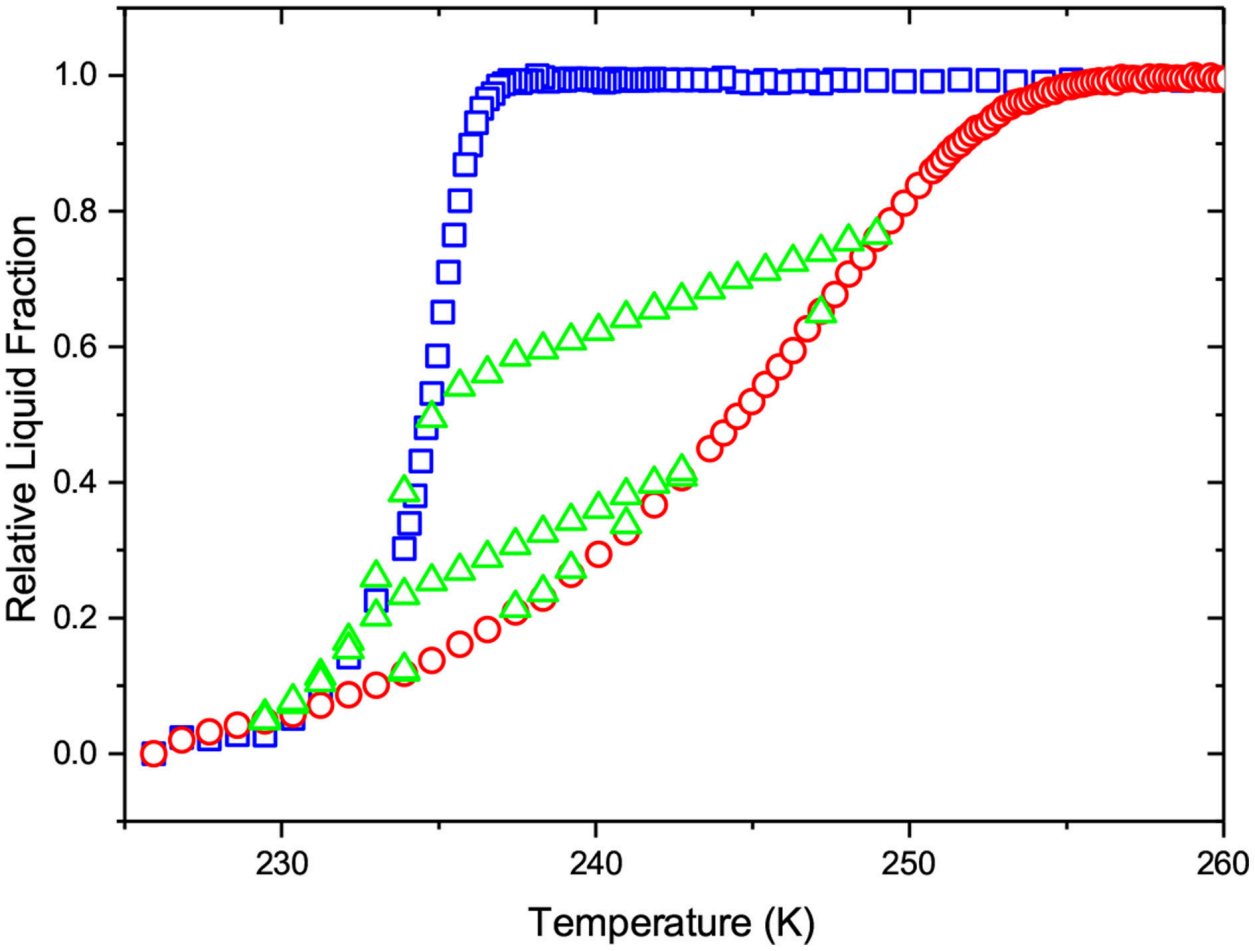
The freezing and melting behaviors of water in mesoporous glass. The figure shows the relative fractions of liquid water obtained on cooling (squares) and warming (circles) as well as two freezing scans (triangles) which were obtained by reverting warming to cooling upon incomplete melting of the intra-pore ice.

Figure 5 shows the relative fractions of liquid water in the porous glass sample as a function of temperature. The boundary freezing and melting transitions exhibit a pronounced Vycor-like behavior with an asymmetric hysteresis loop. Often, such strong asymmetry is associated with pore blocking rendering freezing relatively sharp transition in materials with strong geometric disorder. According to this scenario, ice invasion from the pore openings is delayed until the leading ice front may pass through the smallest pore. The latter occurs when the temperature is decreased to the equilibrium transition temperature of the neck. However, in the present case this scenario fails. While the steepness of the freezing transition still originates from strong disorder and pore blocking, the freezing transition is triggered not by bypass of the necks at their equilibrium transition temperatures, but by homogeneous nucleation of ice in strongly supercooled water.

It is well-known that water can be supercooled only to *T*_*h*_, ranging from about 232 to 235 K according to different authors, where it spontaneously freezes (Bergman and Swenson, 2000; Moore and Molinero, 2011; Amann-Winkel et al., 2016; Mascotto et al., 2017). If a porous solid contains as part of its pore network small pores with the pore sizes below about 3 nm, freezing in these pores does not exhibit typical features of the first-order phase transitions. While the formation of the solid phase is still observed, the process of solidification is rather gradual with decreasing temperature and occurs at temperatures notably below *T*_*h*_ (Morishige and Kawano, 1999). Thus, the presence of such small pores will prohibit the ice invasion at *T* ≥ *T*_*h*_. In the larger pores, however, water cannot sustain the liquid state below *T*_*h*_ and freezes spontaneously at this temperature (Mascotto et al., 2017). This results in a sharp freezing transition around *T*_*h*_. It should be noted that the experimental freezing transition is not step-like, but smeared over a finite temperature range. We believe that this is caused by a pore size dependency of the homogeneous nucleation temperature, similar to that evidenced for the cavitation pressure (Rasmussen et al., 2010). For ice nucleation this phenomenon remains, however, poorly explored.

Besides the closeness of the freezing transition to *T*_*h*_, a stronger support for the homogeneous ice nucleation mechanism is provided by the shapes of the scanning transitions. Their main feature is that they cross the boundary freezing curve. This behavior is typically observed for materials composed of a collection of single pores. However, if this indeed would be the case, the shape of the hysteresis loop would not exhibit a strong asymmetry. On the other hand, in geometrically disordered mesoporous materials the scanning freezing curves should merge at the lower closure point of the hysteresis loop (Kondrashova et al., 2010; Petrov and Furó, 2011). Hence, crossing of the boundary freezing curve in the presence of structural disorder, as observed in Figure 5, can be explained only by the occurrence of the homogeneous ice freezing. If the latter would not be the case, the freezing transition would be more gradual and symmetric with the melting one and the scanning curves would indeed close at the lower closure point.

**Figure 6:**
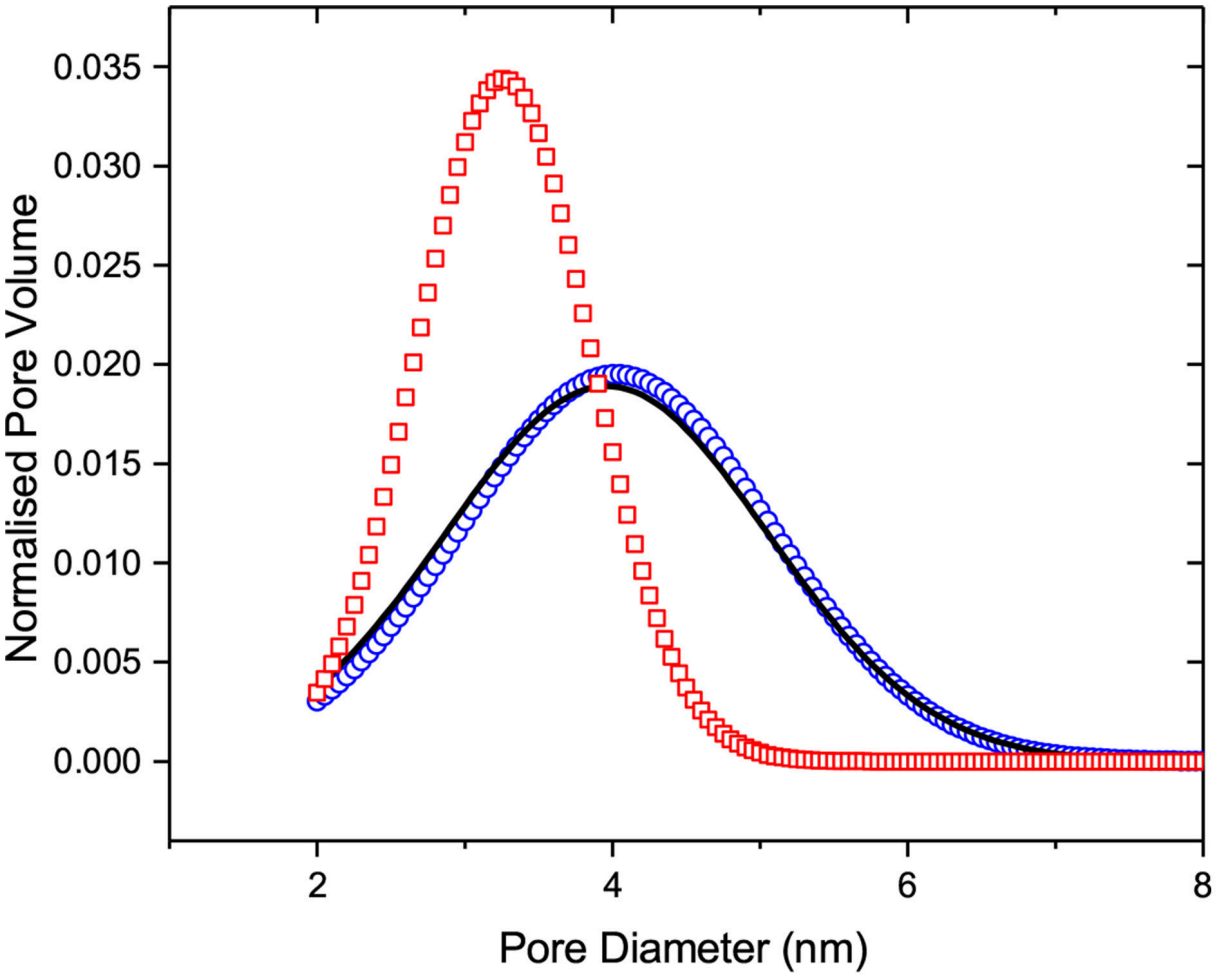
The normalized pore size distribution functions obtained from the melting transition (i) by applying IPM and assuming that melting is the equilibrium (circles) or metastable (squares) transition and (ii) by applying SCPM (line).

**Figure 7:**
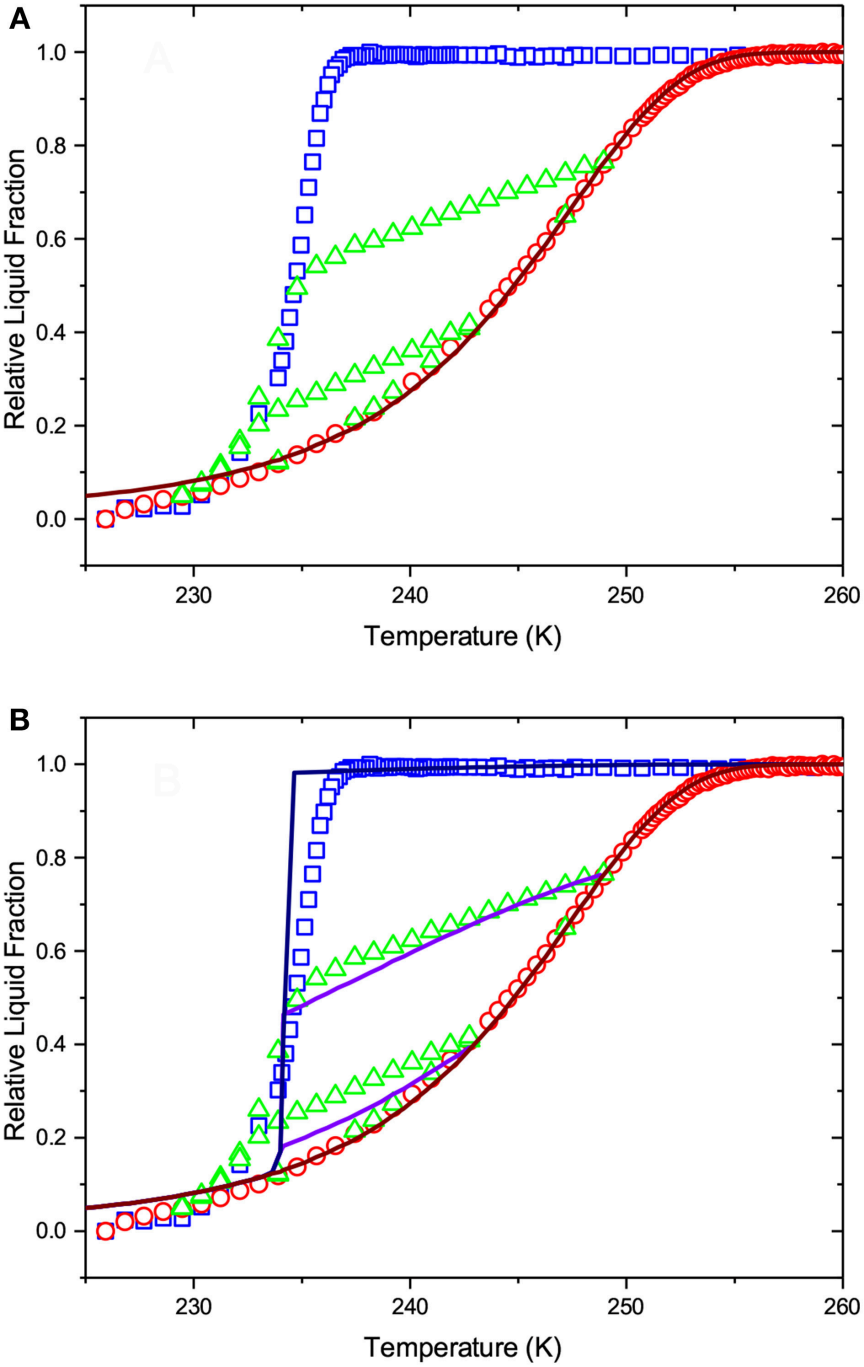
The lines show (i) the best fit of Equation (1) to the boundary melting transition and (ii) the freezing transition and freezing scans as predicted by IPM on the basis of the pore size distribution obtained from the melting transition. Both melting and freezing are assumed to occur at thermodynamic equilibrium. **(A)** shows freezing in independent pores, while in **(B)** pore blocking and homogeneous nucleation are implemented for the freezing transition. The symbols are the experimental data of Figure 5.

Let us now analyze the experimental data in a more quantitative way. First, we apply IPM to see how it correlates with the experimental findings. The main drawback of IPM is that one needs to assume a transition mechanism for a particular transition considered. For the melting transition, most frequently used for the structural analysis of porous solids, an *a priori* assumption whether it occurs at equilibrium or is controlled by metastable liquid bridging needs to be made. Let us first consider the equilibrium melting scenario. In this case, Equation (1) combined with the equilibrium kernel shown in Figure 2A is applied. Let us select a normal PSD with the lower cut-off at 2 nm and fit Equation (1) to the melting transition by varying the distribution width σ and average pore diameter *d*_*a*_ (see PSD obtained in Figure 6). As demonstrated by Figure 7, this procedure yields a reasonably good fit, except a minor discrepancy at low temperatures. The latter is most probably caused by the nuclear magnetic relaxation effects in the NMR cryoporometry experiments and have no impact on the subsequent discussion. As a next step, we validate the applicability of IPM by proving whether this PSD obtained from the analysis of the melting transition may also reproduce the freezing and scanning behaviors. Because it is commonly accepted that freezing is the equilibrium transition, IPM will predict freezing being reversible with melting as shown in Figure 7. That means that IPM fails completely if one assumes equilibrium melting and equilibrium freezing.

**Figure 8:**
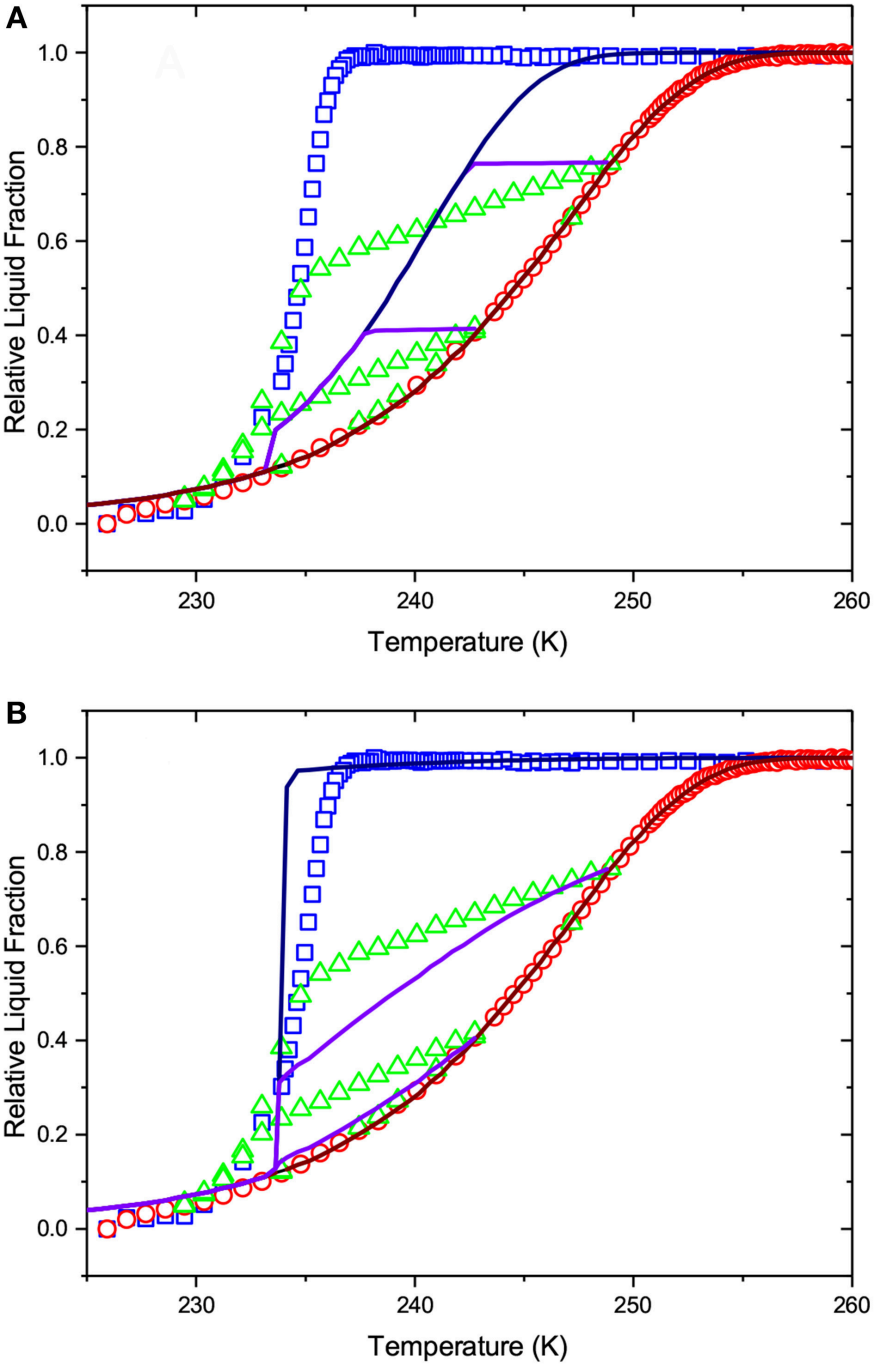
The lines show (i) the best fit of Equation (1) to the boundary melting transition and (ii) the freezing transition and freezing scans as predicted by IPM on the basis of the pore size distribution obtained from the melting transition. Melting is considered as a metastable transition and freezing is assumed to occur at thermodynamic equilibrium. **(A)** shows freezing in independent pores, while in **(B)** pore blocking and homogeneous nucleation are implemented for the freezing transition. The symbols are the experimental data of Figure 5.

By considering an alternative scenario in which melting is assumed to be the metastable transition, we employ Equation (1) with the metastable kernel, Figure 2B. As in the previous case, we use a normal distribution of the pore sizes with the lower cut-off of 2 nm (see the resulting PSD in Figure 6). Once again we obtain a fit accurately reproducing the melting transition, see Figure 8. If now IPM is used to predict freezing and freezing scans on the basis of PSD obtained from melting, it fails again. This proves that IPM cannot capture the phase equilibria in the system studied. These findings reveal in turn that the structural analysis conventionally done using IPM may yield non-correct results.

**Figure 9:**
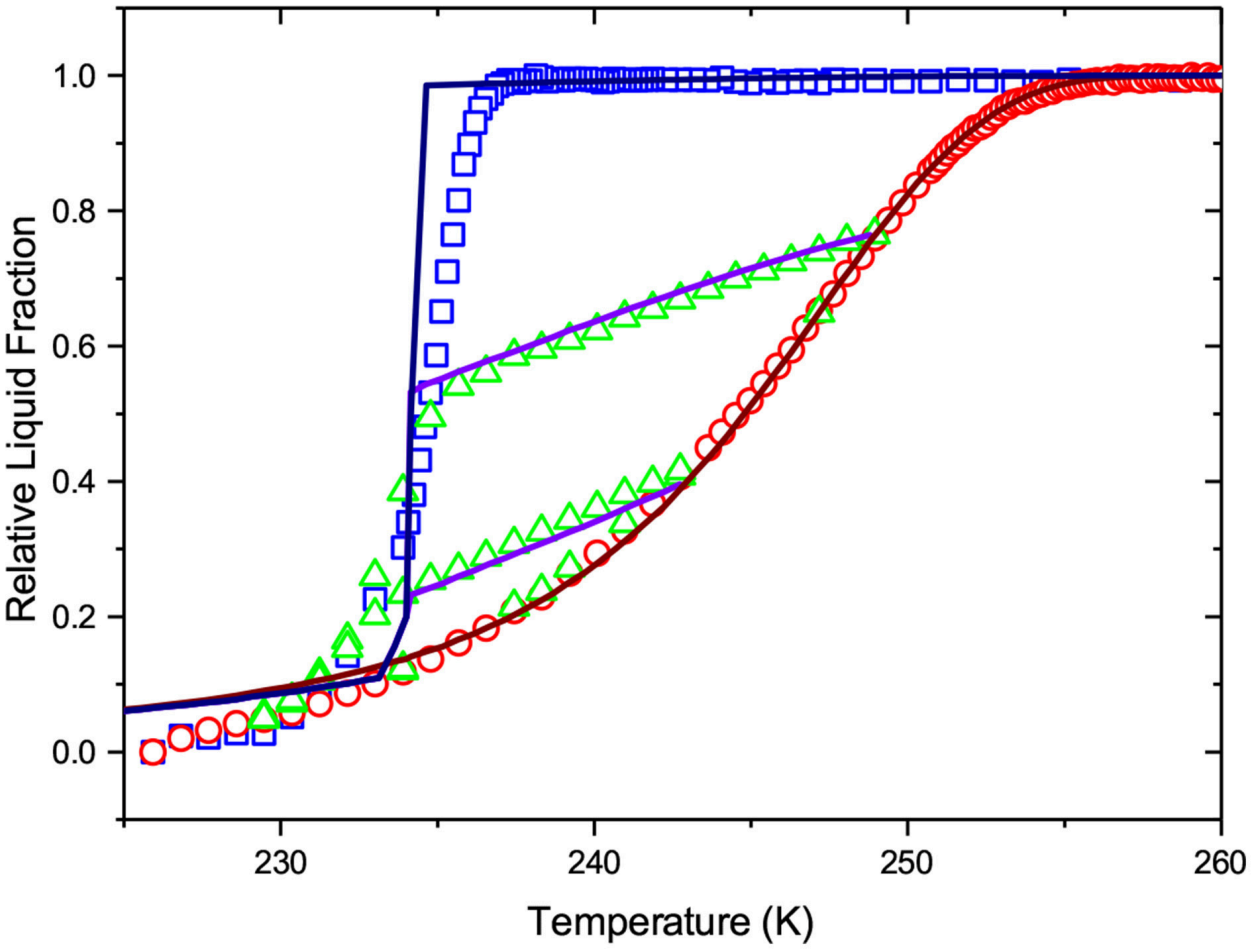
The lines show (i) the best fit of Equation (2) to the boundary melting transition and (ii) the freezing transition and freezing scans as predicted by SCPM on the basis of the pore size distribution obtained from the melting transition. The symbols are the experimental data of Figure 5.

While the IPM framework relies generally on a pre-selection of a transition kernel, SCPM does not require making such assumptions. Moreover, it uses both nucleation and growth kernels with the appropriate weights. Figure 9 demonstrates the application of the same procedure, as described in the two preceding paragraphs, but now using SCPM. It is evident from the figure that, in contrast to both cases based on IPM, now all transitions are satisfactorily reproduced. The respective PSD obtained within SCPM is shown in Figure 6.

It is worth highlighting two interesting observations. First, PSD obtained using SCPM and IPM with the equilibrium melting kernel are found to be very close to each other. This is an indication that melting in this particular material occurs close to equilibrium. This is, in fact, very reasonable if one recalls that the mesoporous glass contains small-sized pores and possesses a substantial structural disorder. Because of the presence of a significant number of small pores with sizes below 3.5 nm and small pores which melt by liquid bridging, the metastability becomes effectively eliminated at already sufficiently low temperatures. Hence, ice in the majority of the pores melts by growth of the already formed liquid domains. The latter occurs at equilibrium, hence the SCPM results become automatically biased toward the equilibrium process.

The second important observation is that, irrespective of the fact that PSD obtained using SCPM and IPM with the equilibrium kernel are nearly identical, the distributions of the liquid and ice phases in the porous glass and its evolution along the melting curve are significantly different for IPM and SCPM. This is proven by Figures 7B, 8B showing what SCPM, namely the model inherently incorporating pore blocking and homogeneous ice nucleation, would predict for the boundary freezing and freezing scans if the melting branch is treated to follow IPM with the equilibrium and metastable kernels, respectively. Notably, while the freezing transition is still reproduced, the freezing scans are not. The reason is that, for the scanning transitions, the distributions of the ice and liquid domains at the initial point of the scan are decisive. These distributions are different for IPM and SCPM. For this reason, though PSDs delivered by SCPM and by IPM with the equilibrium kernel differ only marginally, only SCPM properly reproduces the scanning curves because it correctly describes the evolution of the liquid and ice domains during melting.

### 5.2. Nitrogen Sorption

**Figure 10:**
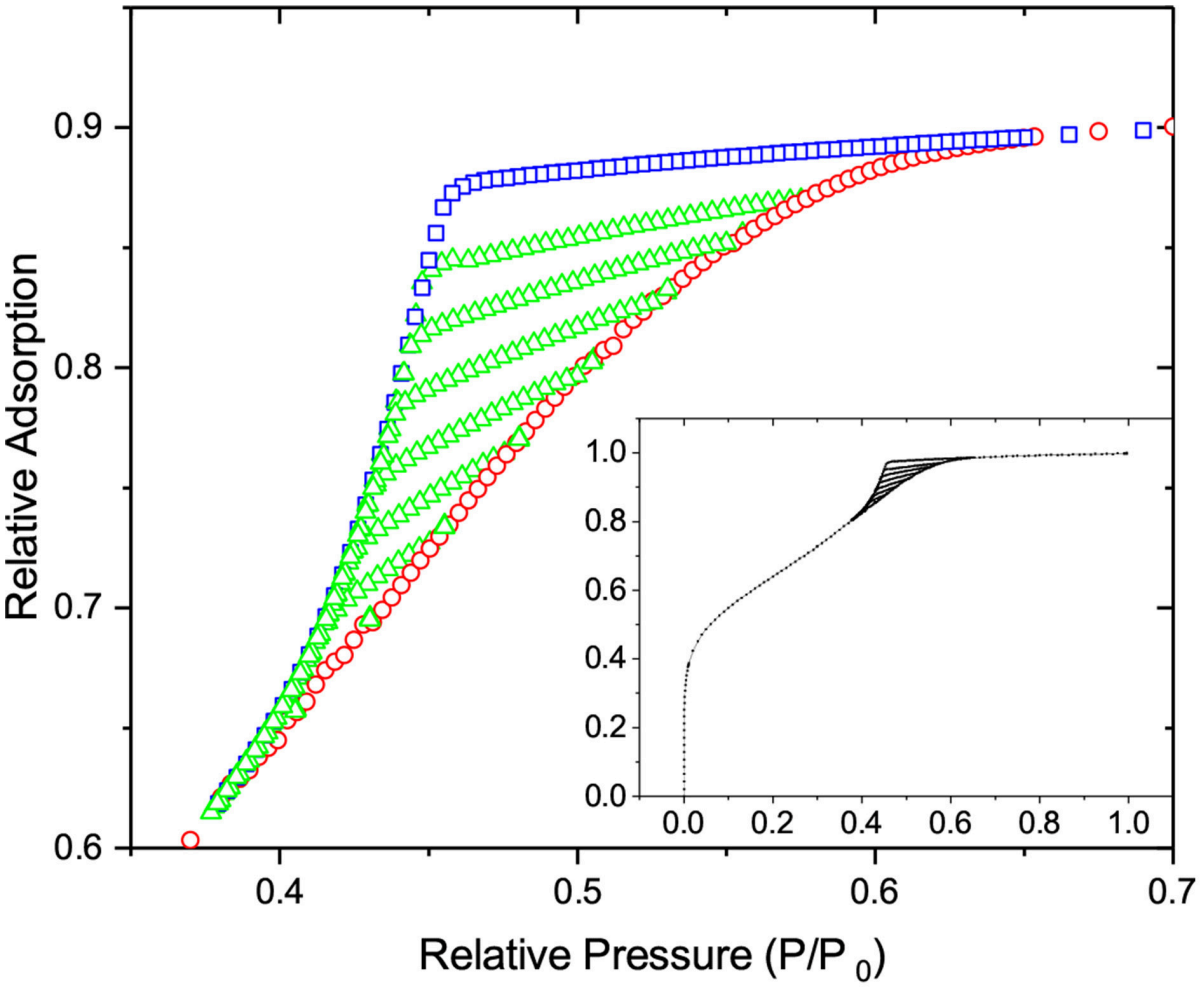
The nitrogen adsorption and desorption behaviors in mesoporous glass at 77 K. The figure shows the relative adsorption obtained on desorption (squares) and adsorption (circles) as well as desorption scans (triangles) which were obtained by reverting adsorption to desorption upon incomplete adsorption. The inset shows the same data, but for the entire range of pressures.

The entire discussion presented for freezing and melting in the preceding section directly applies for adsorption and desorption as well. In this section, we recapitulate shortly the main points. First of all, the nitrogen adsorption isotherm shown in Figure 10 reveals the occurrence of mesoporosity. The well-pronounced asymmetry of the hysteresis loop is an indication of strong disorder with the steeper desorption branch caused by strong pore blocking. The family of the desorption scanning curves, all crossing the boundary desorption curve, indicates that the desorption transition is triggered by cavitation and not by the onset of gas percolation. In the latter case, the desorption scans would close at the lower closure point of the hysteresis.

**Figure 11:**
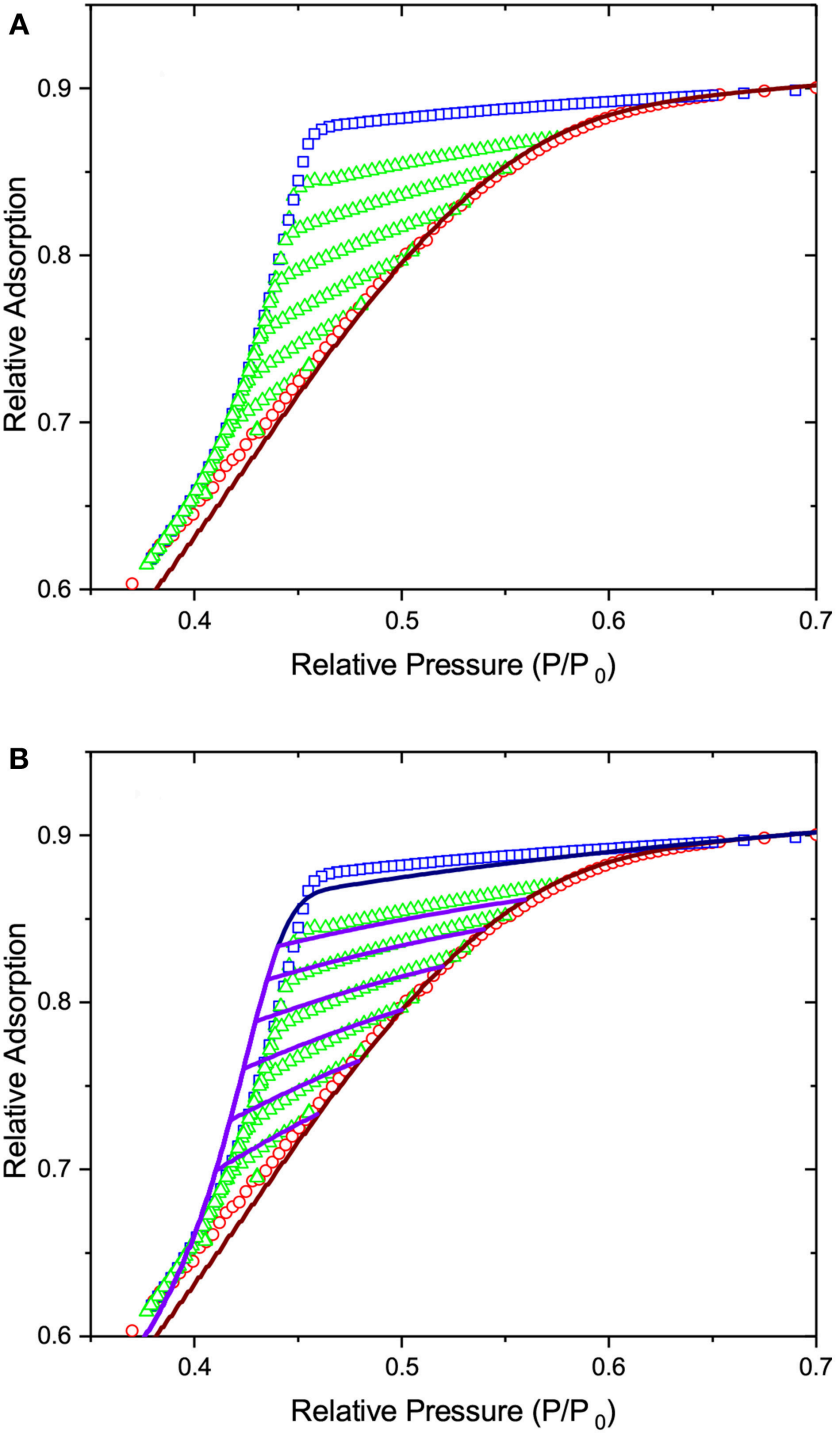
The lines show (i) the best fit of Equation (1) to the adsorption transition and (ii) the desorption transition and desorption scans as predicted by IPM on the basis of the pore size distribution obtained from adsorption. Both adsorption and desorption are assumed to occur at thermodynamic equilibrium. **(A)** shows desorption in independent pores, while in **(B)** pore blocking and cavitation are implemented for the desorption transition. The symbols are the experimental data of Figure 10.

**Figure 12:**
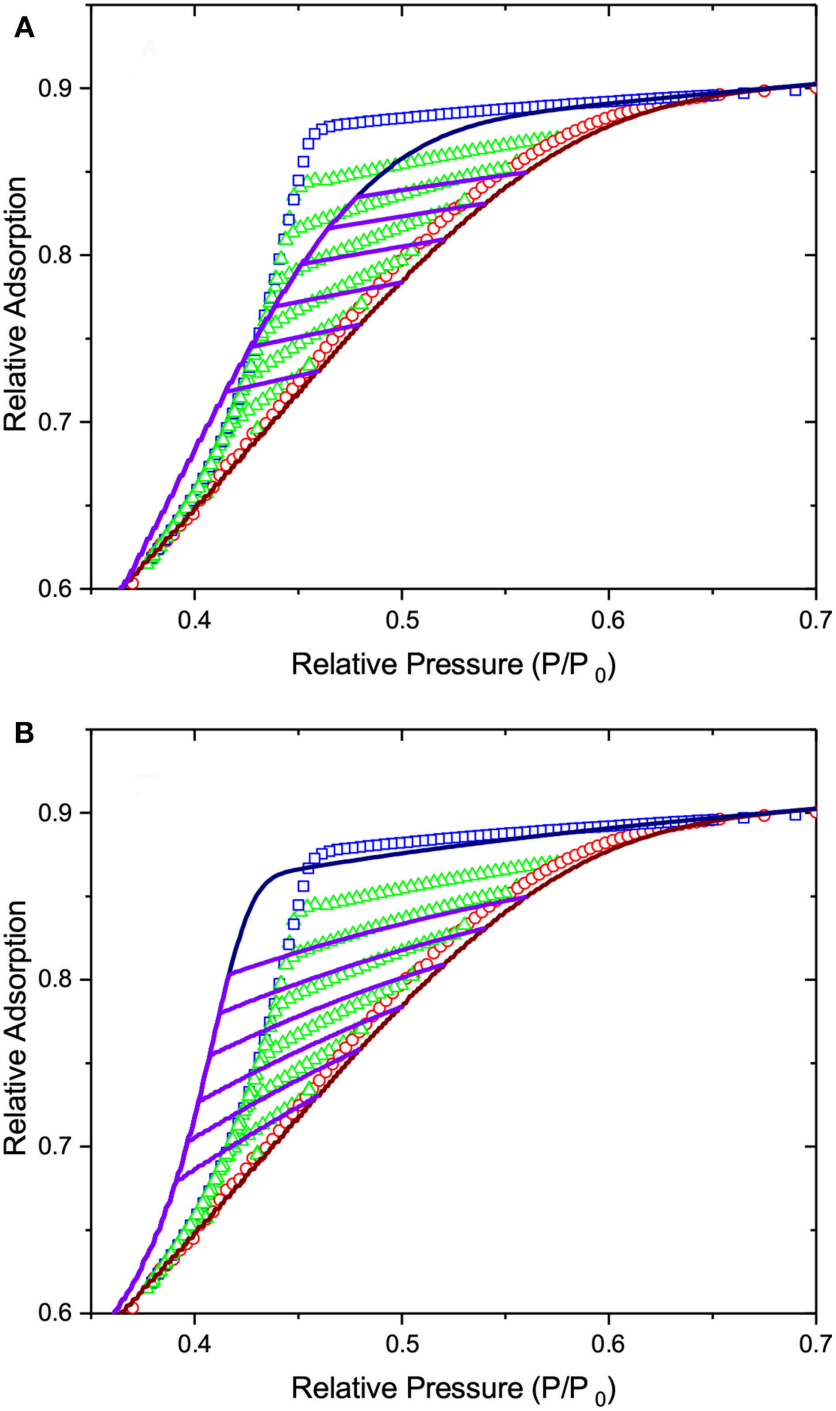
The lines show (i) the best fit of Equation (1) to the adsorption transition and (ii) the desorption transition and desorption scans as predicted by IPM on the basis of the pore size distribution obtained from adsorption. Adsorption is considered as the metastable transition and desorption is assumed to occur at thermodynamic equilibrium. **(A)** shows desorption in independent pores, while in **(B)** pore blocking and cavitation are implemented for the desorption transition. The symbols are the experimental data of Figure 10.

**Figure 13:**
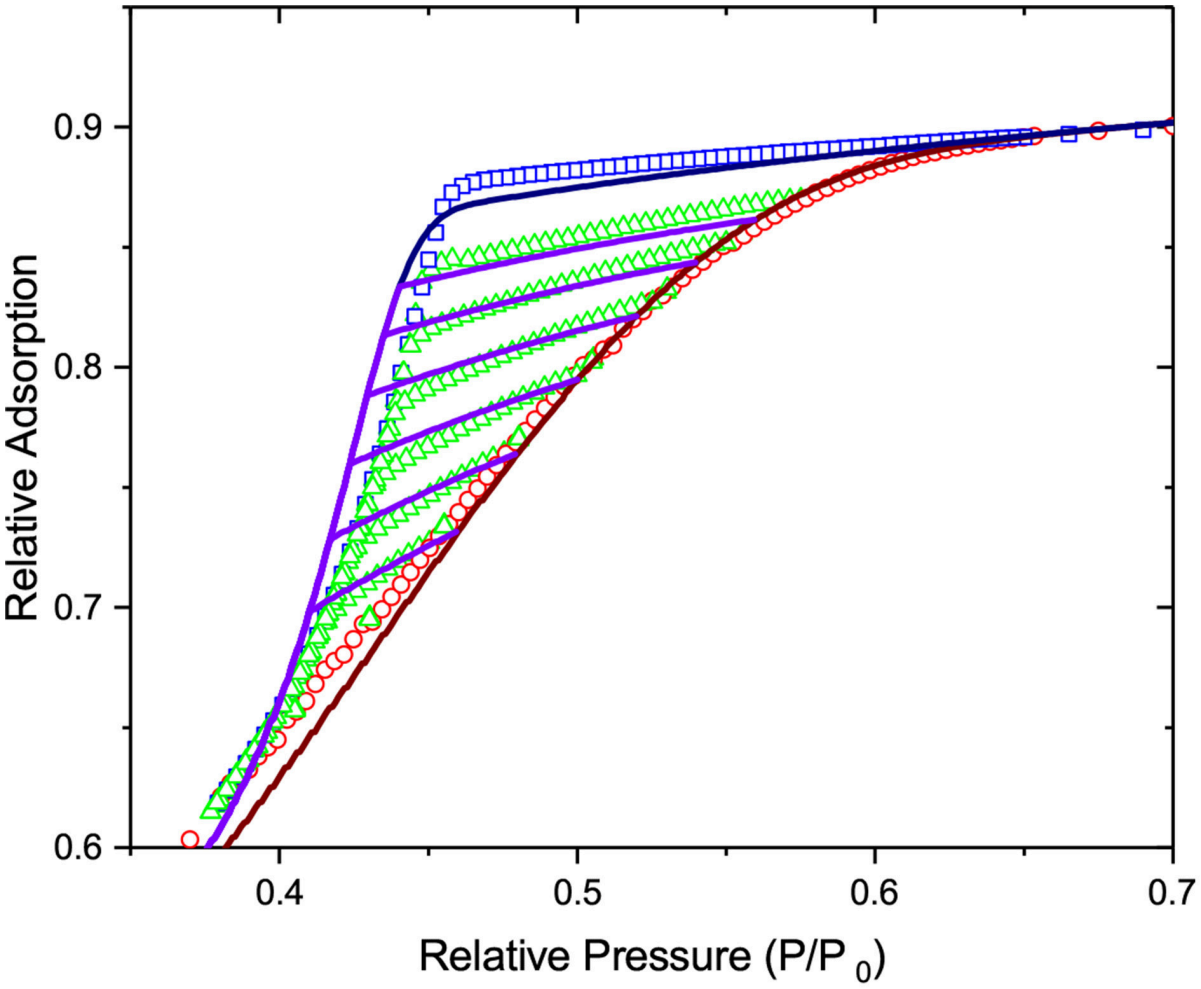
The lines show (i) the best fit of Equation (2) to the adsorption transition and (ii) the desorption transition and desorption scans as predicted by SCPM on the basis of the pore size distribution obtained from adsorption. The symbols are the experimental data of Figure 10.

On considering the performances of IPM and SCPM, the same scenario as for freezing and melting is evident. Thus, as shown by the data of Figure 11, IPM with equilibrium adsorption and desorption kernels predicts the reversibility of the adsorption and desorption isotherms, which is not observed in the experiment. At the same time, as shown in Figure 12, IPM with metastable adsorption and with equilibrium desorption kernels also fails to describe the experimental data. In both cases, the lack of pore blocking in IPM renders the desorption transition false. In contrast to IPM, SCPM, as shown in Figure 13, describes very accurately (within the accuracy of the transition kernels used in this work) all transitions including the scanning behavior without making any *a priori* assumptions on the transition mechanisms.

**Figure 14:**
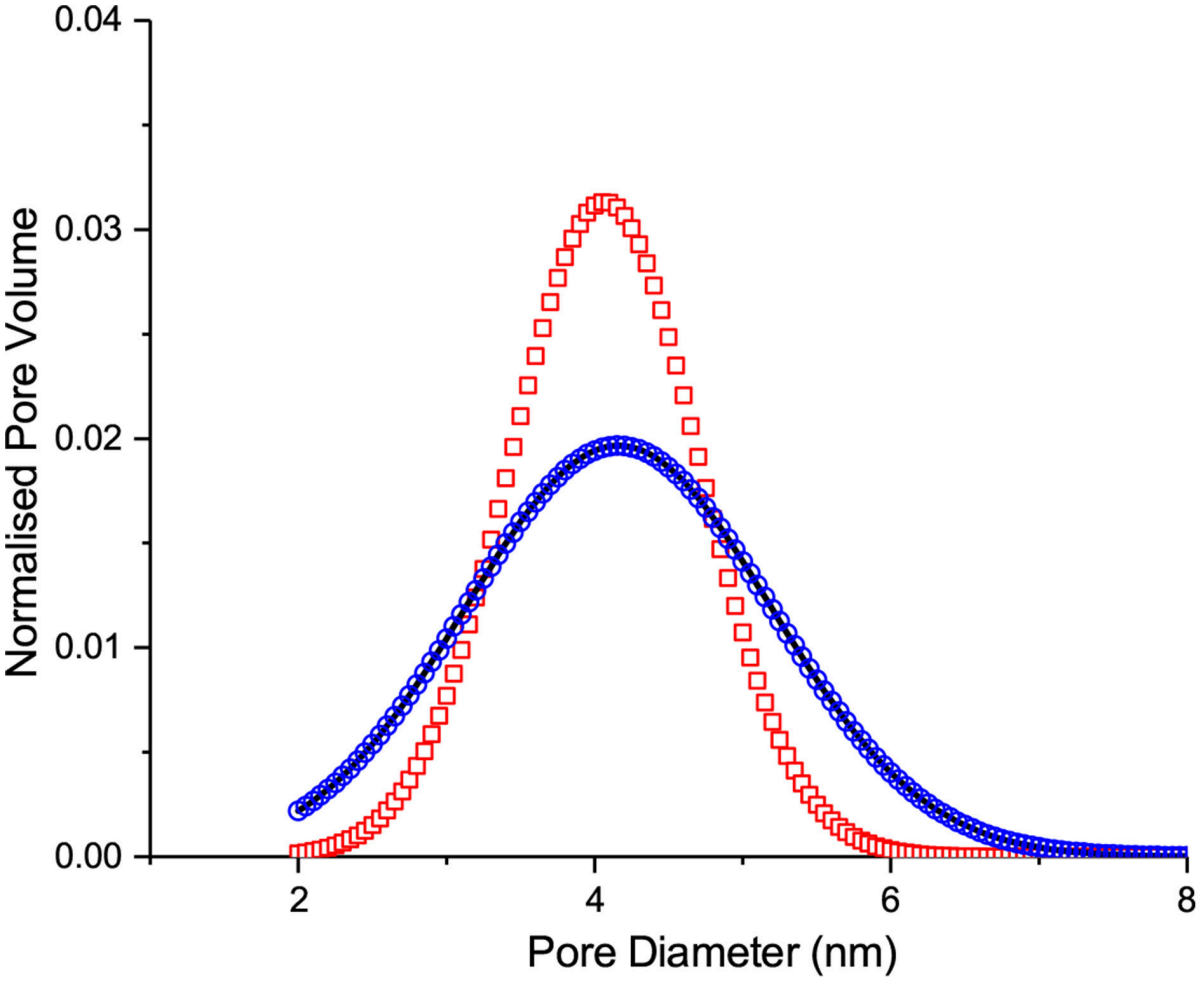
The normalized pore size distribution functions obtained from the adsorption transition (i) by applying IPM and assuming that adsorption is the equilibrium (circles) or metastable (squares) transition and (ii) by applying SCPM (line).

It is interesting to compare PSDs delivered by IPM with equilibrium and metastable adsorption kernels as applied to the adsorption transition with PSD obtained using SCPM. They are shown in Figure 14 and found to be nearly identical. Once again, this demonstrates that even if IPM may be used to obtain reliable PSDs for selected porous materials, it still fails in predicting correct liquid-gas distributions along the transition lines.

## 6. Conclusions

Notable progress made over the last decades in the understanding of fluid behavior in ordered porous solids has made two important impacts. It led to a substantial improvement of the structure characterization techniques for mesoporous solids based on the measurements of phase transitions, especially of gas sorption. On the other hand, by better understanding of single-pore systems it has now become possible to address more complex phenomena arising from network effects. In this work, we demonstrate the first application of the recently developed serially connected pore model for a detailed analysis of the pore structure of a mesoporous glass with highly disordered pore network. The model uses the recent advances in the description of the single-pore materials in terms of the transition kernels and supplements them with the additional mechanisms which are effective in network structures. We show that, in contrast to commonly used independent domain models, the new model reproduces self-consistently all features of the phase equilibria seen in the experiments. The robustness of the model is further supported by the fact that it delivers identical results for both solid-liquid and liquid-gas transitions in the material studied.

**Figure 15:**
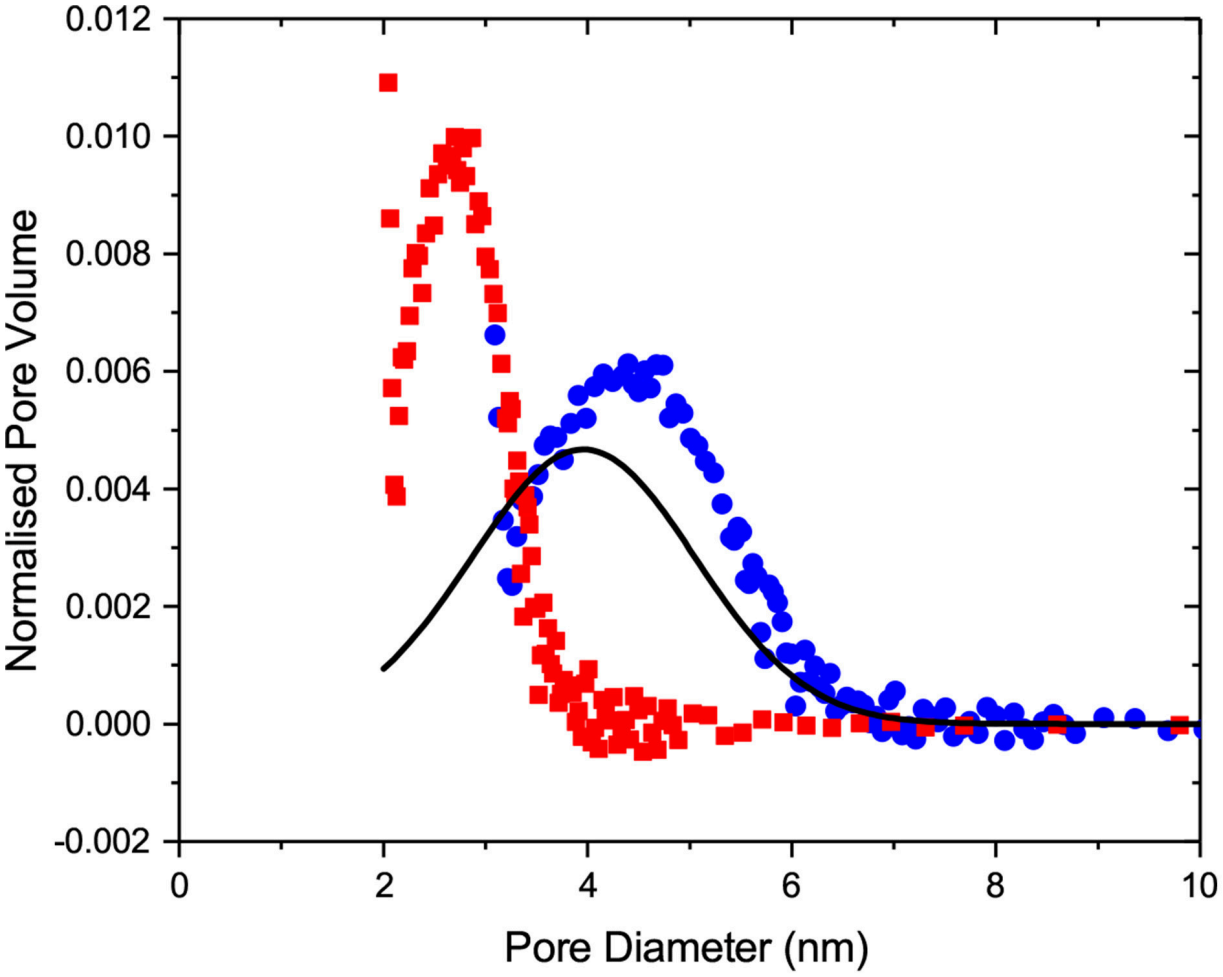
The normalized pore size distribution functions obtained from the melting cryoporometry data using the modified Gibbs-Thompson equation and the conventional approach as introduced in Strange et al. (1993) by assuming that melting is the equilibrium (circles) or metastable (squares) transition and by applying SCPM (line).

On considering the cryoporometry studies, the commonly used procedure to relate the pore sizes with the measured transition curves is based on the application of the advanced Gibbs-Thompson equation, in which a correction for the thickness of the non-frozen liquid-like layers is made. Typically, this thickness is taken to be constant over the entire range of temperatures. In order to improve the analysis (i) by considering the variation of the non-frozen layers thicknesses with temperature, (ii) by capturing the effect of thermodynamic fluctuations diminishing metastabilities with decreasing pore sizes, and (iii) to be consistent with the way of analysis done for gas sorption, we have now introduced the transition kernels for different freezing and melting mechanisms. Within the framework of the independent pore model, the melting and freezing transitions can now be accurately described using a general cryoporometry equation. The latter is an analog of the general sorption isotherm, Equation (1), in which the sorption kernels are simply replaced by the ones for freezing and melting. Furthermore, the introduction of the kernels also ensured that all equations obtained for the serially connected pore model can now be directly applied for the analysis of freezing and melting in the same way as done for gas adsorption and desorption. In this way, as demonstrated by Figure 15, a notable improvement of the structural analysis is achieved.

In the porous glass studied in this work, the average pore size is relatively small, namely of about 4 nm. Hence, a small deviation of the pore sizes in this region leads to substantial changes in the transition points. Thus, with the resulting width of the distribution of about 2 nm, one may classify this material—in the context of the first-order phase transitions—as having strong geometric disorder. On the other hand, in this range of pore sizes, strong impact of thermodynamic fluctuations exists. This leads to the fact that the metastable and equilibrium transition kernels do not differ strongly. Hence, the pore size distributions obtained from the adsorption and melting branches by applying the independent domain model with equilibrium and metastable branches also do not differ notably. The same is valid for the serially connected pore model because it essentially intermixes the two kernels. On considering the accuracy of the structural analysis based solely on the evaluation of adsorption or melting transitions, both independent pore model with equilibrium kernel and serially connected pore model may equally be applied. In this specific range of the pore sizes and of the distribution width, the transitions are strongly biased to occur close to equilibrium, because metastabilities in small pores are effectively eliminated by thermodynamic fluctuations and metastabilities in the larger pores are effectively eliminated by the formation of the liquid domains in small pores. With increasing average pore size, however, metastabilities will start to play an increasingly important role and the good agreement between the two models, as observed in the material studied, will not hold anymore.

In this work, we have further performed the consistency check for different models by inspecting how these models are capable of reproducing not only one transition, but the whole family of the transitions. It turned out that even if the adsorption and melting transitions could be reproduced in the frame of two models using nearly identical pore size distributions, only the serially connected pore model was able to reproduce also the desorption and freezing and scanning transitions. The underlying reason for that is the inclusion of the cooperativity effects resulting from pore-to-pore interconnectivity. In particular, the serially connected pore model is proven to correctly reproduce the sequences of filling and emptying (or similarly melting and freezing) events along the pore spaces, while the independent pore models fail significantly. Once again, all these effects will become more pronounced with increasing average pore size. But already the case study presented here for a small pore size material proves the potentials of the serially connected pore model for structure determination in geometrically disordered porous solids. An important point is that this model yields a perfect agreement for the structural information obtained using two different experimental methods, namely, gas sorption and cryoporometry. To the best of our knowledge, it is the first study where such good quantitative agreement is demonstrated. As a final remark, the present study also highlights the importance of the scanning behavior for improving the structure analysis methods.

## Data Availability

All datasets generated for this study are included in the manuscript and/or the supplementary files.

## Author Contributions

HRNBE performed the cryoporometry experiments and all data analyses and compiled the transition kernels for melting and freezing. DS and RV developed the SCPM theory. AH and DE synthesized the material. SK performed the adsorption experiments and discussed the measurements with MF. All authors discussed the results. RV and DE coordinated research and wrote the manuscript.

### Conflict of Interest Statement

The authors declare that the research was conducted in the absence of any commercial or financial relationships that could be construed as a potential conflict of interest.
